# Comparative Genomics of *Pediococcus pentosaceus* Isolated From Different Niches Reveals Genetic Diversity in Carbohydrate Metabolism and Immune System

**DOI:** 10.3389/fmicb.2020.00253

**Published:** 2020-02-26

**Authors:** Jie Jiang, Bo Yang, R. Paul Ross, Catherine Stanton, Jianxin Zhao, Hao Zhang, Wei Chen

**Affiliations:** ^1^State Key Laboratory of Food Science and Technology, Jiangnan University, Wuxi, China; ^2^School of Food Science and Technology, Jiangnan University, Wuxi, China; ^3^International Joint Research Center for Probiotics and Gut Health, Jiangnan University, Wuxi, China; ^4^APC Microbiome Ireland, University College Cork, Cork, Ireland; ^5^Moorepark Teagasc Food Research Centre, Cork, Ireland; ^6^National Engineering Research Center for Functional Food, Jiangnan University, Wuxi, China; ^7^Wuxi Translational Medicine Research Center and Jiangsu Translational Medicine Research Institute Wuxi Branch, Wuxi, China; ^8^Beijing Advanced Innovation Center for Food Nutrition and Human Health, Beijing Technology and Business University, Beijing, China

**Keywords:** *Pediococcus pentosaceus*, comparative genomics, genetic diversity, carbohydrate utilization, bacteriocin, CRISPR/Cas, prophage

## Abstract

*Pediococcus pentosaceus* isolated from fermented food and the gastrointestinal tracts of humans and animals have been widely identified, and some strains have been reported to reduce inflammation, encephalopathy, obesity and fatty liver in animals. In this study, the genomes of 65 *P. pentosaceus* strains isolated from human and animal feces and different fermented food were sequenced and comparative genomics analysis was performed on all strains along with nine sequenced representative strains to preliminarily reveal the lifestyle of *P. pentosaceus*, and investigate the genomic diversity within this species. The results reveal that *P. pentosaceus* is not host-specific, and shares core genes encoding proteins related to translation, ribosomal structure and biogenesis and signal transduction mechanisms, while its genetic diversity relates mainly to carbohydrate metabolism, and horizontally transferred DNA, especially prophages and bacteriocins encoded on plasmids. Additionally, this is the first report of a type IIA CRISPR/Cas system in *P. pentosaceus.* This work provides expanded resources of *P. pentosaceus* genomes, and offers a framework for understanding the biotechnological potential of this species.

## Introduction

*Pediococcus pentosaceus* has a long history of use in the food industry due to its ability to produce antimicrobial agents (Porto et al., 2017). Various strains of *P. pentosaceus* have been reported to produce bacteriocins, including pediocin PA-1, pediocin ST18, pediocin-A and pediocin ACCEL (Piva and Headon, 1994; Wu et al., 2004; Miller et al., 2005; Todorov and Dicks, 2005). These bacteriocins are responsible for the ability of *P. pentosaceus* to inhibit pathogenic and spoilage bacteria during food/beverage storage (Porto et al., 2017), such as *Listeria monocytogenes* (Komora et al., 2020) and *Oenococcus oeni* (Diez et al., 2012). *P. pentosaceus* may also exhibit inhibitory activity toward other *P. pentosaceus* strains differing in antimicrobial production as well as toward *P. acidilactici* and *Lactobacillus plantarum*, which may share niches with *P. pentosaceus* (Luc de Vuyst, 1994). In addition to its antibacterial ability, other functions of *P. pentosaceus* have also attracted much attention, such as regulating immunity (Silva et al., 2017; Bamba et al., 2018), and improving the growth and final meat quality as well as gut micro-environment of chicken and fish (Dawood et al., 2016; Adel et al., 2017; Chen et al., 2017). Recently, some strains were reported to alleviate toxicity from vital organs such as liver and intestine caused by cadmium (Cd) exposure in a murine model (Dubey et al., 2019). Other studies have reported that exopolysaccharide (EPS) fractions from *P. pentosaceus* stimulate IFN-c-primed macrophages and primary splenocytes to induce immune responses and improve cyclophosphamide induced immunosuppression in mice (Shin et al., 2016; Yasutake et al., 2016).

Despite the potential of *P. pentosaceus* to function as a probiotic, only a limited number of strains have been isolated from various habitats thus limiting comparative analysis. *P. pentosaceus* has been reported to be naturally associated with plants, fruits, plant fermentations and meat materials. A few isolates have been detected in the gastrointestinal (GI) tracts of poultry and ducks as well as in freshwater prawns (Jetten et al., 2015). Recently a novel strain was isolated from the rumen of goat (Ladha and Jeevaratnam, 2018). However, most of the strains available in the public database (NCBI) have been isolated from fermented food and the natural environment. Only three *P. pentosaceus* strains isolated from the human GI tract or fecal samples have been released and available for analysis (*P. pentosaceus* SS1-3, CGMCC7049 and MGYG-HGUT-02367).

Predicting and expanding the probiotic potential of *P. pentosaceus* is of much interest. The genomic diversity within the genus *Pediococcus* was investigated using RAPD-PCR and PFGE (Simpson et al., 2002). Furthermore, seventeen strains of *P. pentosaceus*, isolated from food matrices, have been studied to develop a multilocus sequence typing (MLST) method of genomic characterization and revealed the genotypic diversity of penocin operons (Martino et al., 2013), In 2014, *P. pentosaceus* LI05 (CGMCC 7049) was isolated from the human gastrointestinal tract and compared to three representative food-borne strains through genomic analyses, and was found to exhibit unique physiological and metabolic properties (Lv et al., 2014). However, current research on the genomic diversity of *P. pentosaceus* has only been conducted with a few strain and single niches. However, to further explore the probiotic capabilities of this species, more genomic analyses are essential.

The aim of this work was to sequence the genomes of 65 *P. pentosaceus* strains isolated from different niches in China, particularly human feces which has been rarely reported; and to perform comparative genomic analysis on all 65 strains to investigate the genomic diversity among the species, to reveal correlations between genotypes and phenotypes as well as to analyze potential habitat adaptation, which will guide further mining of probiotic traits in this species and enable its engineering.

## Materials and Methods

### Ethics Statement

This study was approved by the Ethics Committee in Jiangnan University, China (SYXK 2012-0002). All the fecal samples from healthy persons were for public health purposes and these were the only human materials used in present study. Written informed consent for the use of their fecal samples were obtained from the participants or their legal guardians. All of them conducted health questionnaires before sampling and no human experiments were involved. The collection of fecal sample had no risk of predictable harm or discomfort to the participants. And sampling of homemade fermented food and domestic animals were all consented by owners.

### Culturing of Strains, Genome Sequencing and Data Assembly

Sixty-five strains were previously isolated from human feces, dairy products and fermented vegetables from different regions in China, which are listed in Table 1. All the strains had been identified as *P. pentosaceus* using 16s rRNA sequencing. Strains were cultured in de Man, Rogosa and Sharpe (MRS) agar (Difco, BD Biosciences, Franklin Lakes, NJ, United States) for 48 h at 37°C in an anaerobic chamber (AW400TG, Electrotek Scientific Ltd., West Yorkshire, United Kingdom). The draft genomes of all the strains were sequenced via Illumina Hiseq × 10 platform (Majorbio BioTech Co, Shanghai, China), which generated 2 × 150 bp paired-end libraries and constructed a paired-end library with an average read length of about 400 bp. The reads were assembled by SOAPde-novo and local inner gaps were filled by using the software GapCloser (Luo et al., 2012). For each bacterium, assemblies were constructed using K-mers from 21 to 41, and the other assembly parameters are listed in Supplementary Table S1. And nine publicly available genomes (*P. pentosaceus* ATCC25745, *P. pentosaceus* DSM20336, *P. pentosaceus* IE3, *P. pentosaceus* KCCM40703, *P. pentosaceus* NBRC3182, *P. pentosaceus* SL4, *P. pentosaceus* SRCM100194, *P. pentosaceus* SRCM100892, *P. pentosaceus* WIKIM20) from NCBI GenBank database^1^ were used for further genomic comparison in the work.

**TABLE 1:** The information of 74 *P. pentosaceus* strains.

Strains	Source	Genome size (Mb)	GC (%)	CDS no.	Accession number	References
HN22-4	Human Feces	1.86	37.11	1881	SAMN13139753	This work
HN39-6	Human Feces	1.78	37.34	1801	SAMN13139754	This work
HN25-2	Human Feces	1.94	37.11	1981	SAMN13139755	This work
HN34-1	Human Feces	1.81	37.12	1809	SAMN13139756	This work
FHNXY65L1	Duck Feces	1.89	37.25	1941	SAMN13139757	This work
HN26M2	Human Feces	1.77	37.31	1801	SAMN13139758	This work
FHNFQ2L2	Human Feces	1.77	37.15	1778	SAMN13139759	This work
FHNFQ13L3	Human Feces	1.75	37.24	1738	SAMN13139760	This work
FHNFQ51L1	Human Feces	1.94	37.16	1927	SAMN13139761	This work
FHNFQ30L2	Human Feces	2.11	37.16	2170	SAMN13139762	This work
FHNXY71L2	Chicken Feces	1.70	37.18	1703	SAMN13139763	This work
FHNXY69M10	Chicken Feces	1.80	37.25	1815	SAMN13139764	This work
FHNXY66L3	Duck Feces	1.75	37.31	1727	SAMN13139765	This work
FHNXY64M2	Chicken Feces	1.84	37.2	1851	SAMN13139766	This work
FHNFQ52L1	Human Feces	1.80	37.33	1796	SAMN13139767	This work
FHNFQ36L4	Human Feces	1.84	37.18	1867	SAMN13139768	This work
JHNZZ2M34	Emptins	1.70	37.37	1692	SAMN13139769	This work
FJLHD57M2	Duck Feces	1.79	37.24	1772	SAMN13139770	This work
FNXYCHL77L4	Duck Feces	1.97	37.14	1969	SAMN13139771	This work
FSDQZ42L1	Human Feces	2.05	37.04	2096	SAMN13139772	This work
FSDQZ12L1	Dog Feces	1.87	37.37	1875	SAMN13139773	This work
FSDLZ40M4	Human Feces	1.85	37.07	1850	SAMN13139774	This work
FSDLZ47M8	Human Feces	1.85	37.25	1856	SAMN13139775	This work
FSDHZD5L7	Human Feces	1.83	37.07	1852	SAMN13139776	This work
FSDHZD6L3	Human Feces	1.83	37.1	1828	SAMN13139777	This work
FSCDJY13M1	Human Feces	1.90	37.16	1920	SAMN13139778	This work
FSCPS3M1	Human Feces	1.81	36.92	1836	SAMN13139779	This work
FSCPS31M1	Human Feces	1.81	36.94	1836	SAMN13139780	This work
FSCPS12M2	Human Feces	1.84	37.17	1846	SAMN13139781	This work
FSCPS15M1	Human Feces	1.89	37.07	1919	SAMN13139782	This work
FSCCD1M2	Human Feces	1.86	37.23	1844	SAMN13139783	This work
FSCPS27L1	Human Feces	1.77	36.99	1776	SAMN13139784	This work
FSCCD11L5	Human Feces	1.87	37.32	1846	SAMN13139785	This work
FSCDJY60M2	Human Feces	1.97	37.25	1998	SAMN13139786	This work
FSCPS35L3	Human Feces	1.80	36.91	1831	SAMN13139787	This work
FSCPS86L4	Chicken Feces	1.84	37.17	1845	SAMN13139788	This work
FSCCD23L1	Human Feces	1.92	37.09	1935	SAMN13139789	This work
FSCCD15L1	Human Feces	1.95	37.03	2019	SAMN13139790	This work
FSCDJY41L1	Human Feces	1.89	37.22	1904	SAMN13139791	This work
FSCPS24L2	Human Feces	1.92	37.16	1956	SAMN13139792	This work
FSCDJY49L1	Human Feces	1.82	36.92	1807	SAMN13139793	This work
FSCDJY57L3	Human Feces	1.85	37.26	1857	SAMN13139794	This work
FWXBH44M2	Human Feces	1.94	37.19	1953	SAMN13139795	This work
FWXBH2M1	Human Feces	1.89	37.13	1900	SAMN13139796	This work
FYNLJ92L1	Chicken Feces	1.85	37.16	1892	SAMN13139797	This work
FYNLJ84L2	Goose Feces	2.01	37.31	2061	SAMN13139798	This work
FYNLJ90L2	Chicken Feces	1.93	37.02	2013	SAMN13139799	This work
FYNLJ66M1	Human Feces	1.91	37.08	1943	SAMN13139800	This work
FYNLJ46M4	Human Feces	1.79	37.08	1759	SAMN13139801	This work
DYNDL40M2	Milk	1.87	37.24	1897	SAMN13139802	This work
DYNDL46L1	Dairy Fan	1.90	37.17	1932	SAMN13139803	This work
DYNDL35L7	Dairy Fan	1.92	37.18	1998	SAMN13139804	This work
DYNDL5L1	Dairy Fan	1.93	37.81	2026	SAMN13139805	This work
DYNDL19L1	Dairy Fan	1.89	37.88	1962	SAMN13139806	This work
DYNDL26L10	Dairy Fan	1.82	38.01	1865	SAMN13139807	This work
DYNDL53M5	Dairy Fan	1.82	38.11	1867	SAMN13139808	This work
DYNDL62M3	Dairy Fan	1.86	37.99	1932	SAMN13139809	This work
DYNDL69M8	Dairy Fan	1.90	37.88	2010	SAMN13139810	This work
FYNLJ19L1	Human Feces	1.88	37.79	1944	SAMN13139811	This work
FYNLJ37M7	Human Feces	1.85	37.93	1860	SAMN13139812	This work
FCQHC27L4	Chicken Feces	1.88	37.18	1865	SAMN13139813	This work
FCQNA15L8	Human Feces	1.75	37.06	1754	SAMN13139814	This work
FCQNA17L4	Human Feces	1.96	37.28	1955	SAMN13139815	This work
VCQYC5144M12	Pickle	1.90	37.19	1885	SAMN13139816	This work
FCQNA36M2	Human Feces	1.90	37.14	1915	SAMN13139817	This work
ATCC25745	Plant	1.83	37.40	1814	SAMN02598525	Diep et al., 2006
SL4	Kimchi	1.79	37.30	1784	SAMN02603952	Dantoft et al., 2013
WIKIM20	Kimchi	1.83	37.26	1789	SAMN04017317	Lee et al., 2016
SRCM100892	Food	2.00	37.27	2078	SAMN07126152	Jeong et al., 2015
SRCM100194	Food	1.87	37.38	1849	SAMN07224387	Jeong et al., 2015
KCCM40703	Sake Mash	1.76	37.20	1767	SAMN06447729	Unpublished
IE3	Diary	1.80	37.20	1775	SAMEA2272526	Midha et al., 2012
DSM20336	Agricultural Product	1.74	37.30	1725	SAMN02797817	Sun et al., 2015
NBRC3182	Fermented Milk	1.83	37.00	1866	SAMD00093675	Unpublished

### Average Nucleotide Identity (ANI) Values

ANI between any two genomes was calculated using python script^2^ (Richter and Rossello-Mora, 2009) and the resulting matrix was clustered and visualized using TBtools heatmap software (Chen et al., 2018).

### Comparative Genomic Analysis

All the protein sequences were compared using all-against-all alignments (Altschul et al., 1990). Then the orthologous genes were clustered using OrthoMCL version 1.4 (Dongen, 2000; Li et al., 2003; Chen et al., 2007). Those genes which were common among all the 74 *P. pentosaceus* strains were defined as orthologous genes presented at least once in each genome assayed; and those genes consisting of those presenting in some but not each genome made up the variable or dispensable genes; all of the orthologous genes were categorized in predicted functional groups based on COG (Clusters of Orthologous Groups) assignments (Tatusov et al., 2000).

### Pan-Core Genome

For all genomes used in this study, pan-genome calculation was performed using PGAP-1.2.1 (Zhao et al., 2012), in order to calculate the total gene repertoire encountered in the newly sequenced *P. pentosaceus* and the degree of overlap and diversity with respect to other publicly available genomes. The pan-genome of 73 genomes generated a total number of 139,271 gene families for this species (due to the depth of sequencing *P. pentosaceus* IE-3 was excluded). Core-genome and specific genome were analyzed by the CD-HIT cluster analysis similar to protein software (Fu et al., 2012). Among them, amino acids had 50% pairwise identity and 0.7 length difference cut-off threshold (Harris et al., 2017).

### Phylogenetic Analysis

All the genomic DNA were translated to protein sequences by EMBOSS-6.6.0 (Rice et al., 2000). Orthologous genes were used to construct phylogenetic trees using the python script^3^ and the tree was modified using Evolgenius^4^ (Zhang et al., 2012).

### Multiple Genome Alignments

To further compare the genomes clustered in the same clade in the phylogenetic tree, and to find out the potential evolutionary relationship among strains isolated from same region but different samples, the multiple genome alignments were performed using MAUVE software, as a tool to check for synteny amongst large blocks of genomic sequences (Rissman et al., 2009; Darling et al., 2010).

### Whole Genome Comparison

BLAST Ring Image Generator (BRIG) (Alikhan et al., 2011) was used to show a genome wide visualization of coding sequences identity among all the 74 strains, taking ATCC25745 as a reference genome.

### CRISPR Identification and Characterization of Isolated Strains

The Clustered Regularly Interspaced Short Palindromic Repeats (CRISPR) regions and CRISPR-associated (Cas) proteins were identified by CRISPRCasFinder with default parameters (Couvin et al., 2018)^5^, and the CRISPR subtypes designation was based on the signature of Cas proteins (Makarova et al., 2015b). Protospacer adjacent motifs (PAM) were identified from flanking sequences of protospacers (∼10 nucleotides[nt]) by CRISPRTarget (Biswas et al., 2013) with default parameters^6^.

### Bacteriocin Identification

All of the strains were assessed for the presence of bacteriocin operons by BAGEL4 and the domains of bacteriocin were determined using BLASTP analysis against the non-redundant protein databases created by BLASTP based on NCBI (van Heel et al., 2018).

### Genotype/Phenotype Association Applied to Carbohydrate Metabolism

All the genomes were annotated by HMM method in HMMER-3.1, and the carbohydrate active enzymes involved were analyzed by CAZY database^7^ (Besemer et al., 2001; Lombard et al., 2014). An *in silico* evaluation of the role of specific genes associated with carbohydrate unitization was performed using a gene-trait matching (GTM) analysis based on the association between the presence or absence and (if present) the number of gene families, and growth/non-growth phenotype of the 65 *P. pentosaceus* strains.

The capabilities of 65 *P. pentosaceus* strains to metabolize 23 carbohydrate substrates as sole carbon source were determined. Briefly, different carbohydrates including D-lactose, alpha-lactose, L-arabinose, salicin, D-trehalose, D-cellobiose, Xylo-oligosaccharide (XOS), D-ribose, D-galactose, D-fructose, D-mannose, D-Fucose, D-maltose, L-rhamnose, D-raffinose, D-mannitol, D-melezitose, D-sorbitol, 2′-fucosyllactose (2FL), sodium gluconate, D-xylose, sucrose, Fructooligosaccharide (FOS) [Sangon Biotech (Shanghai) Co., Ltd.] were selected for carbohydrate utilization analysis. A 10% (w/v) aqueous stock solution of each carbohydrate was prepared, filtered through a 0.22 μm sterile membrane filter and stored at 4°C prior to use. The utilization assay medium was freshly prepared as for MRS medium (Hyatt et al., 2010), however, glucose was omitted, and bromocresol purple was added into the medium as indicator. After autoclaving and cooling, the sterile carbohydrate stock was added into the medium at 1% final concentration. In order to test the utilization capacity of each strain, after sub-culture twice, a 1% inoculum was inoculated into the test growth medium, each of which was supplemented with a different sugar instead of glucose. Carbohydrate utilization was observed by color change and measured with a microplate reader at OD_600 nm_ (Varioskan Lux, Thermo, MA, United States) after anaerobic culture at 37°C for 24 h (Hyatt et al., 2010). The tests were performed in triplicate.

## Results

### General Genome Characteristics of *P. pentosaceus*

Previously in our laboratory, 65 *P. pentosaceus* strains were isolated from different ecological niches, including human feces, animal feces, fermented vegetables and dairy products (Table 1). In total, 74 *P. pentosaceus* genomes were compared, among which nine genomes were obtained from the NCBI GenBank database (Table 1). The genome size of all the 74 strains ranged from 1.70 Mb for *P. pentosaceus* FHNXY71L2 to 2.11 Mb for *P. pentosaceus* FHNFQ30L2, which followed a normal distribution with an average value of 1.86 Mb. The average percentage G + C content was 37.26%, ranging from 36.91% for *P. pentosaceus* FS35L3 to 35.11% for *P. pentosaceus* DYNDL53M5. The average predicted coding sequences (CDSs) per genome was 1879, ranging from 1692 for *P. pentosaceus* JHNZZ2M34 to 2170 for *P. pentosaceus* FHNFQ30L2.

### Comparative Analyses

**FIGURE 1:**
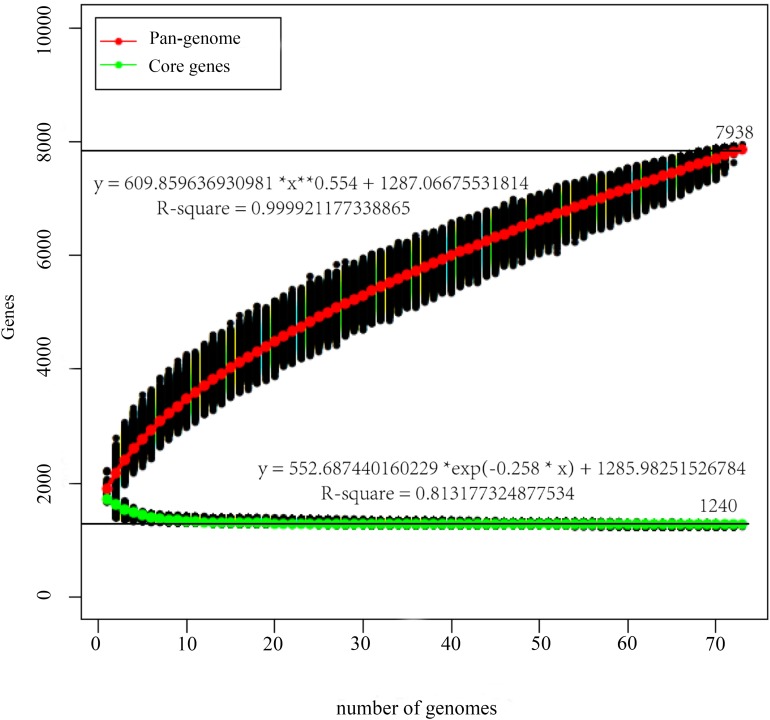
Pan-genome and core-genome of *P. pentosaceus.* The pan-genome plot is represented by the accumulated number of new genes against the number of genomes added. The core-genome plot is represented by the accumulated number of genes attributed to the core-genomes against the number of added genomes. The black line represents the pan-genome and core-genome attributed to the 73 *P. pentosaceus* strains.

**FIGURE 2:**
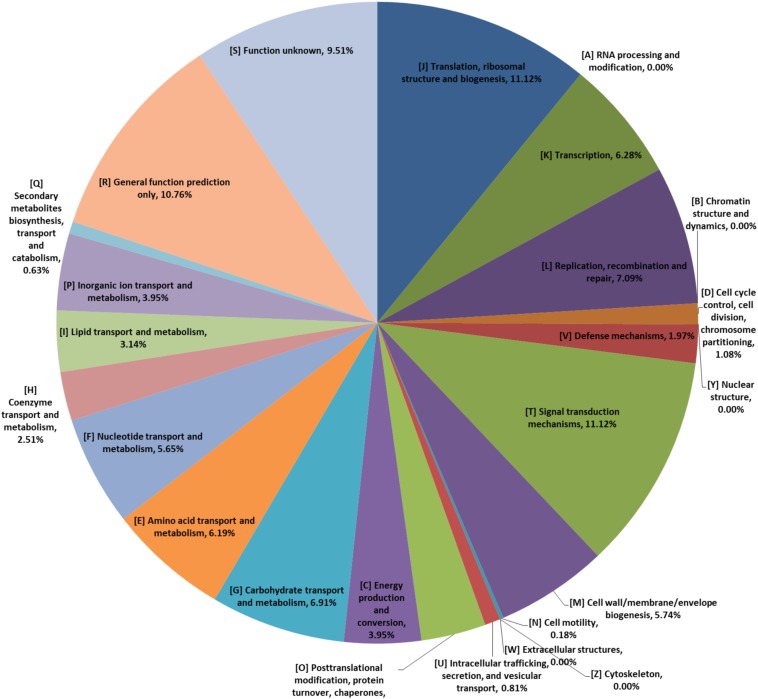
The COG annotation of core-genomes of *P. pentosaceus.*

The pan-genome curve displayed an asymptotic trend with a growth rate of an average of 285 gene families per genome in the first 2 iterations, decreasing to an average of 50 in the final two additions, and generating a total number of 7938 pan genes. Consistently, the core-genome reached a value of 1240 genes at the last iteration (Figure 1). Furthermore, most of those 1240 genes encoded proteins related to translation, ribosomal structure and biogenesis ([J], which were encoded by genes accounting for 11.48% of those genes), signal transduction mechanisms ([T], 11.48%), and general function prediction only ([R], 12.02%) (Figure 2). The trends displayed in the pan-genome indicate that the pan-genome calculation for the included 73 genomes is not yet fully closed, but approaching closure.

**FIGURE 3:**
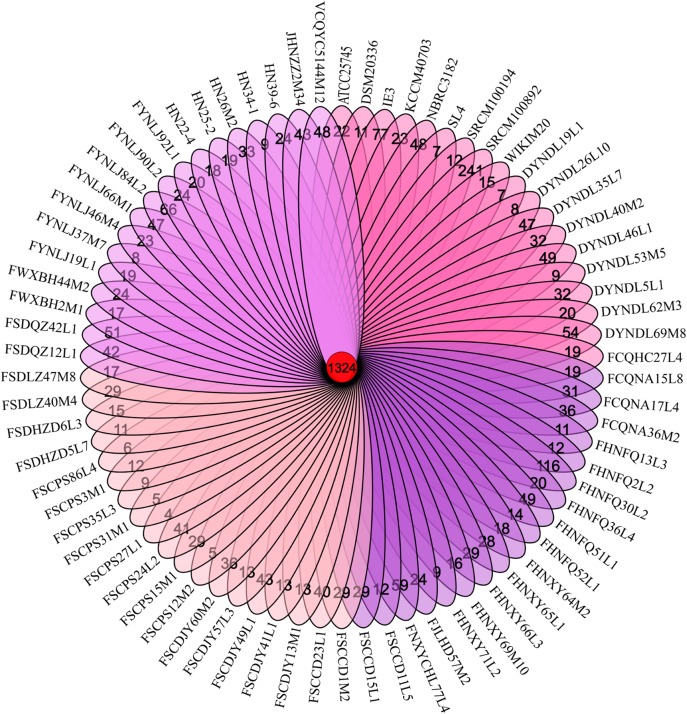
Venn diagram. Venn diagram obtained by MCL clustering displaying core gene families, and unique genes of 74 *P. pentosaceus.*

In order to evaluate the genetic content of the *P. pentosaceus* species, comparative analyses were employed using a BLASTP-mediated approach. Thousand three hundred and twenty four orthologous gene families accounting for 22% of the total gene families were obtained; and the remaining 78% represented variable or dispensable genes. In addition, among all the 4726 variable genes, 2170 unique or strain-specific genes were revealed, of which 241 presented in the *P. pentosaceus* SRCM100892, and 116 in the *P. pentosaceus* FHNFQ30L2 (Figure 3).

### ANI and Phylogenetic Analysis

**FIGURE 4:**
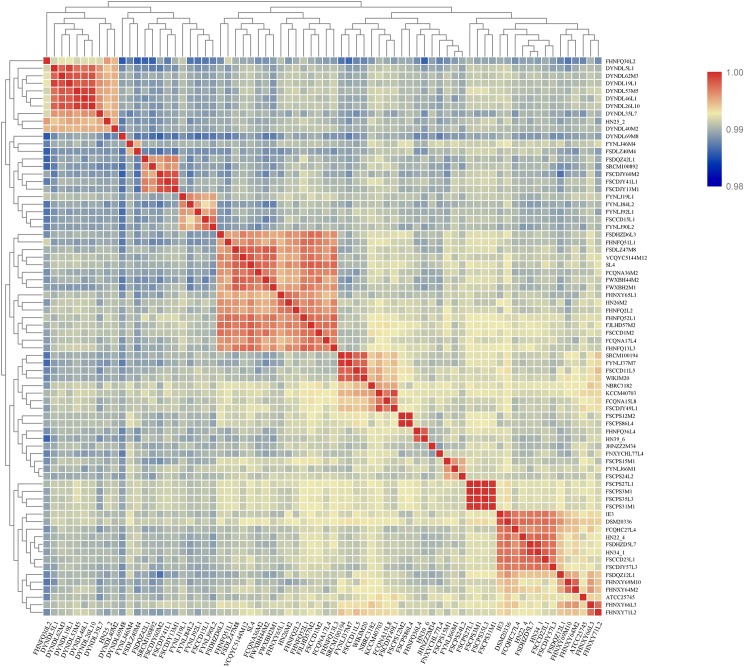
Heatmap showing ANI of *P. pentosaceus* strains. Proposed species cut-off boundary is around 95–96%, showing identity within theses strains.

**FIGURE 5:**
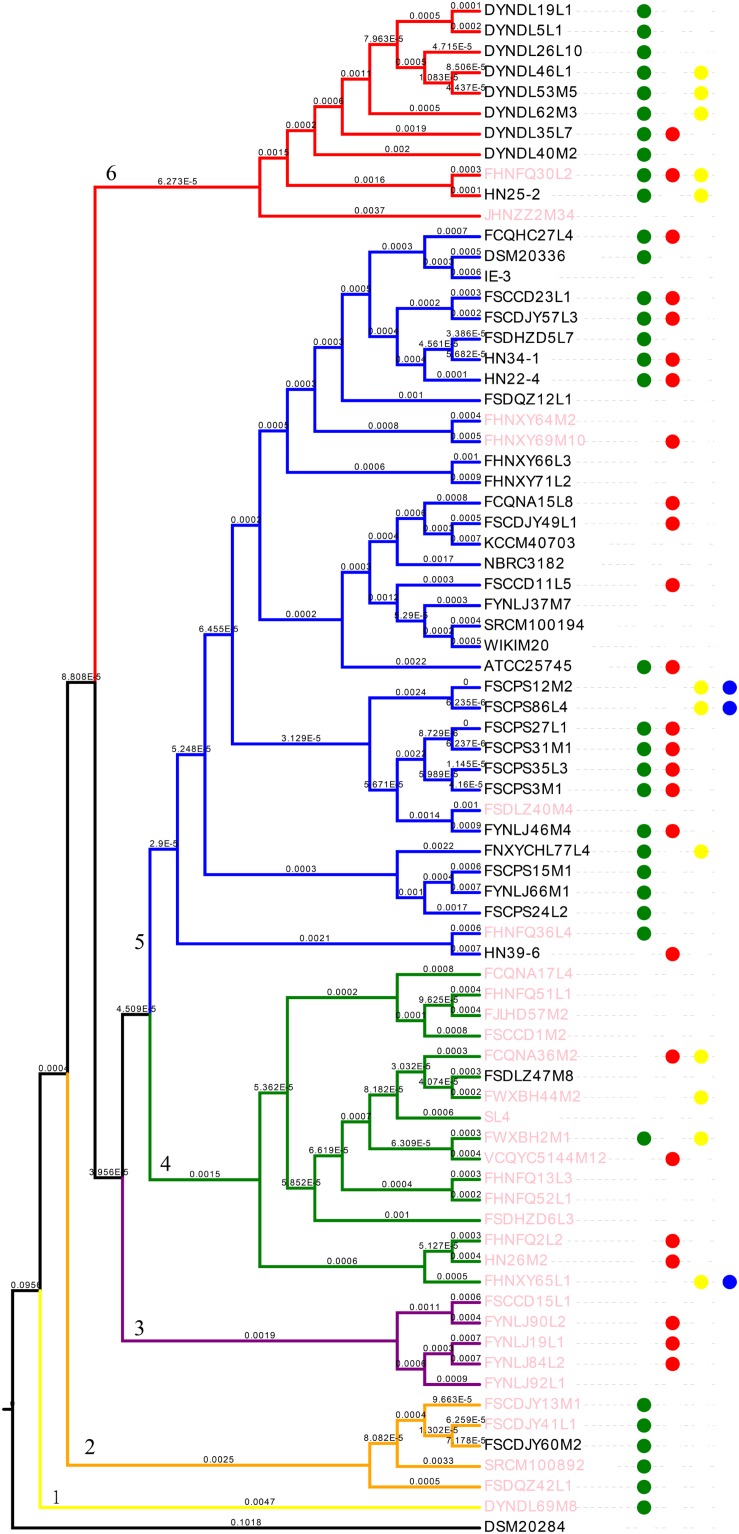
Phylogenetic analysis of *P. pentosaceus.* Phylogenetic supertree showing the relationship among 74 *P. pentosaceus* strains with *P. acidilactici* DSM20284 as outgroup. The tree was grouped in to six clades signed by number 1–6. The dots represented strains that contained the operon of bacteriocins. In specific, red dots represented enterolysin A, green dots represented penocin, yellow dots pediocin, blue dots Bac. Pink font represented strains contained CRISPR/Cas type IIA.

All the strains belonged to *P. pentosaceus*, as evidenced by the ANI value which was above 98% (Valeriano et al., 2019) (Figure 4). For analyzing the phylogenetic relationships among the *P. pentosaceus* isolates, a phylogenetic super-tree was generated based on homologous genes of 74 genomes that constituted the core genome. The resulting phylogenetic tree revealed the presence of six major branches, which was rooted by *P. acidilactici* DSM20284 as an outgroup (Figure 5). The first branch consisted of only one strain isolated from a dairy product, while the sixth branch contained the remaining eight dairy-derived strains with one from Chinese traditional fermented food “emptins” and two strains from human fecal samples. Apart from this, strains were not correlated based on isolation source or sampling region.

### The Multiple Genome Alignments Using MAUVE

**FIGURE 6:**
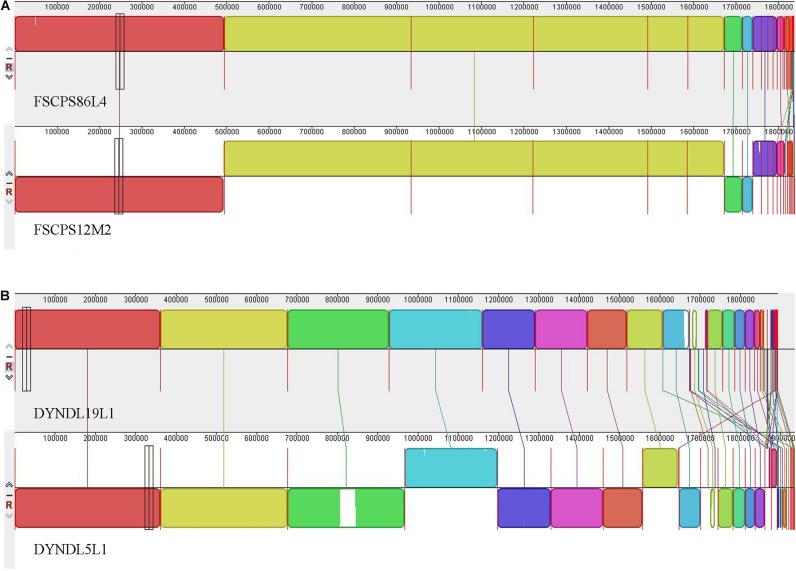
The multiple genome alignment. **(A)** The multiple genome alignment between FSCPS86L4 and FSCPS12M2. **(B)** The multiple genome alignment between DYNDL19L1 and DYNDL5L1.

The multiple genome alignments showed that a high level of synteny exists between FSCPS86L4 and FSCPS12M2 (Figure 6A); FSCPS86L4 was isolated from chicken feces and FSCPS12M2 was isolated from a human fecal sample. Strain DYNDL19L1 had a certain similarity with DYNDL5L1, which were both isolated from homemade fermented dairy products (Dairy fan) from Dali city; a few genes presented in both strains, in some cases with inversions and different positioning within each chromosome (Figure 6B).

### Whole Genome Comparison

**FIGURE 7:**
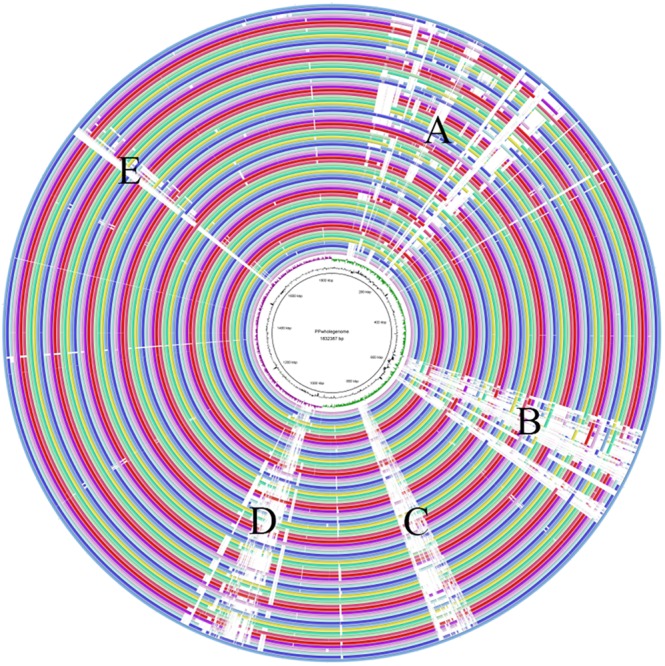
The whole genome comparison. Region A mainly included genes related to defense mechanism, amino acid and carbohydrate transport and metabolism, and cell wall/membrane/envelope biogenesis. Region B related to the cell wall/membrane/envelope biogenesis. Region C and Region D were both phage related. Region E consisted of genes associated with stress response, secondary metabolites biosynthesis and carbohydrate transport and metabolism.

The whole genomes of all 74 *P. pentosaceus* strains were compared with *P. pentosaceus* ATCC 25745 as reference genome using BRIG (Figure 7), where similarity is represented by the solid part of the circle, while variability is represented as a blank space, meaning open reading frames that are present in the reference genome but absent in the remaining genomes (which also correlate to the differences in assembly quality). The variable parts were distributed in five regions. According to the annotation of the referenced genome (Supplementary Table S2), region A mainly includes genes related to defense mechanism, amino acid and carbohydrate transport and metabolism, and cell wall/membrane/envelope biogenesis. Region B relates to the cell wall/membrane/envelope biogenesis as well, while Region C and Region D are both phage related. Region E consists of genes associated with stress response, secondary metabolites biosynthesis and carbohydrate transport and metabolism.

### Carbohydrate Utilization Phenotypic Characterization of *P. pentosaceus*

**FIGURE 8:**
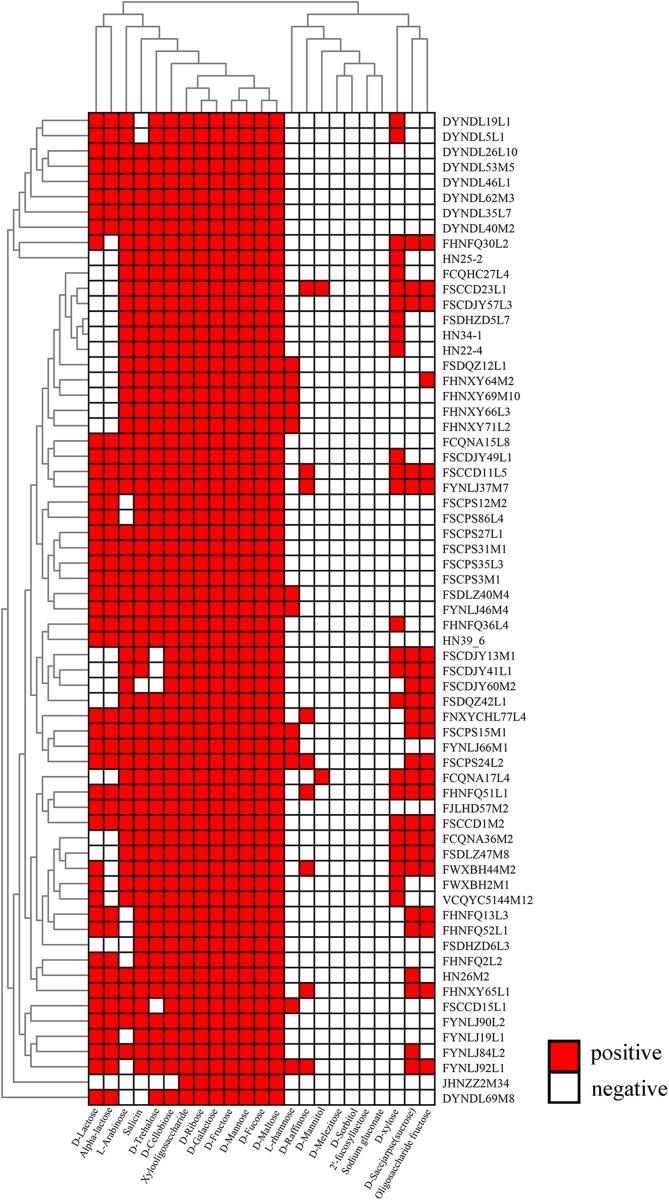
Evaluation of carbohydrate utilization by *P. pentosaceus* strains. Heatmap showing the growth performance of *P. pentosaceus* strains on different carbon sources at 24 h. The vertical axis represents the genomes tested in this study which were clustered according to the phylogenetic tree. The horizontal axis represents 23 carbohydrates used in this test. The color scales represent the degree of *P. pentosaceus* utilizing carbohydrates with white squares (as in blank) represents the inability of strains to metabolize the corresponding sugar and red squares representing the ability of strains to metabolize the corresponding sugar.

All the strains were able to grow in medium containing either D-ribose, D-maltose, D-galactose, D-mannose, D-fructose, and D-fucose as sole carbon source, while none could utilize D-sorbitol, 2′FL and sodium gluconate. The capabilities of the 65 strains to utilize the remaining carbohydrates were variable (Figure 8). The strain JHNZZ2M34 showed the most variable sugar utilization pattern, being the only strain not capable of growing on D-cellobiose. In addition, FSCCD23L1 and FCQNA17L4 were able to grow on D-mannitol which is in contrast to all the other strains tested in this study. Notably, XOS supported the growth of all of the strains tested, whereas the FOS only supported the growth of 23 strains.

### *In silico* Gene-Trait-Matching

**FIGURE 9:**
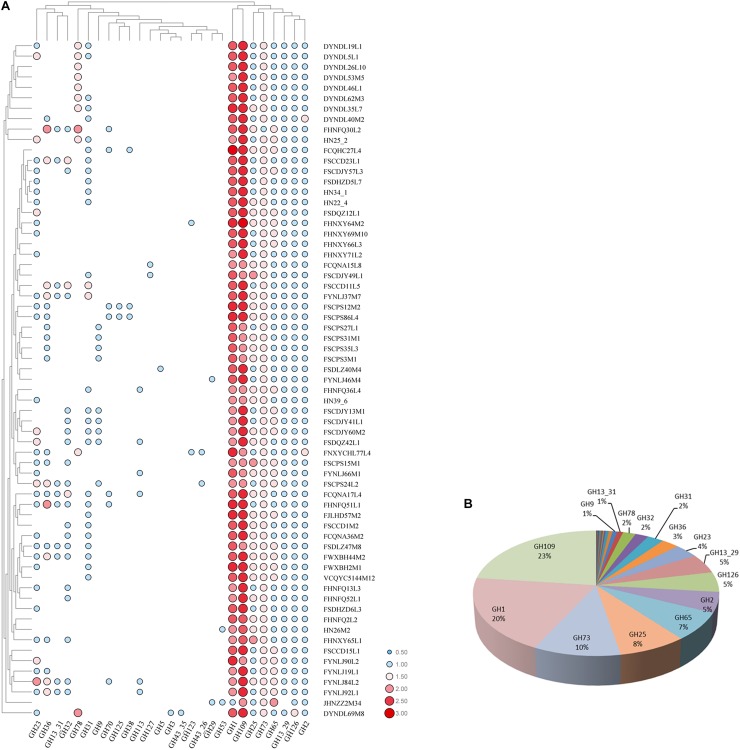
GH families identified in *P. pentosaceus.* **(A)** Heatmap showing the distribution of GH families in 65 *P. pentosaceus* (The number of GH families was log scaled). The vertical axis represents the genomes that harbor GH families which was clustered according to the phylogenetic tree. The horizontal axis represents GH families predicted in *P. pentosaceus*. The color scales represent the number of GH families existed in each genome with white dot (as in blank) represents the absent of matches and red squares representing the highest number of GH families. **(B)** Pie chart indicating the percentage of each GH family identified in *P. pentosaceus* genomes.

Glycosyl hydrolases are the key enzymes that metabolize carbohydrates, and a total number of 27 glycosyl hydrolases were annotated in all 65 strains (Figures 9A,B), of which eight glycosyl hydrolases presented in almost all of the strains, including GH109 (alpha-*N*-acetylgalactosaminidase [EC 3.2.1.49]), GH1 (6-phospho-beta-glucosidase [EC 3.2.1.86]), GH73 and GH25 (lysozyme [EC 3.2.1.17]), GH65 (maltose phosphorylase [EC 2.4.1.8]), GH2 (beta-galactosidase [EC 3.2.1.23]), GH126 (alpha-amylase [EC 3.2.1.-]), and GH13_29 (trehalose-6-phosphate hydrolase [EC 3.2.1.93]). In addition, seven glycosyl hydrolases showed diverse presence in all of the 65 strains, consisting of GH23 (lysozyme type G [EC 3.2.1.17]), GH36 (alpha-galactosidase [EC 3.2.1.22]), GH31 (alpha-xylosidase [EC 3.2.1.177]), GH32 (invertase [EC 3.2.1.26]), GH78 (alpha-L-rhamnosidase [EC 3.2.1.40]), GH13_31 (alpha-glucosidase [EC 3.2.1.20]), and GH9 (endoglucanase [EC 3.2.1.4]). Additionally, the last twelve glycosyl hydrolases only existed in a few strains.

**FIGURE 10:**
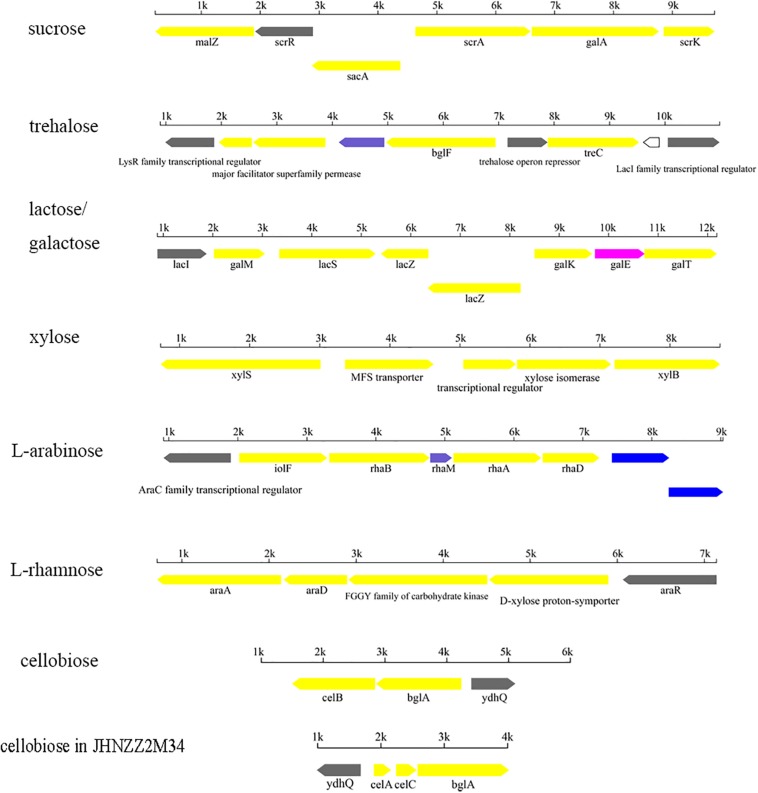
Putative operons for the predicted utilization of carbohydrates in *P. pentosaceus.*

The sequenced *P. pentosaceus* genomes were further analyzed to identify putative operons involved in carbohydrate transport and utilization. Seven putative operons were identified (Figure 10). Metabolism of sucrose in *P. pentosaceus* was linked to GH32, including invertase [EC 3.2.1.26] and beta-fructosidase. In total, 21 strains with genes encoding invertase or beta-fructosidase showed the utilization of sucrose, among which twenty of those strains could utilize FOS. Invertase was encoded by gene *sacA*, along with which alpha-glucosidase [EC 3.2.1.20] (*malZ*) and alpha-galactosidase [EC 3.2.1.22] (*galA*) were potentially co-transcribed with the sucrose operon, however, they might not have a role in the hydrolysis of sucrose or FOS (O’Donnell et al., 2011). Another correlation existed between the presence of the GH13_29 family and D-trehalose utilization, with four exceptions among all the 65 strains. The trehalose operon consisted of *bglF* (PTS transporter subunit IIB and IIC for trehalose), *treC* (trehalose-6-phosphate hydrolase [EC 3.2.1.93], GH13_29) and *lacl* (LysR family transcriptional regulator) (Figure 10).

Beta-galactosidase [EC 3.2.1.23], belonging to the GH2 family, was responsible for metabolism of D-galactose, alpha-lactose, and D-lactose. All the 65 strains containing GH2 were able to grow on D-galactose as sole carbon source, in which 44 strains were able to utilize D-lactose, and 40 strains were able to utilize L-lactose. Notably, all nine strains isolated from dairy products were able to metabolize both D-lactose and L-lactose. The lactose operon involved *lacS* (PTS sugar transporter subunit IIA), *lacZ* (beta-galactosidase) and *lacI* (LacI family transcriptional regulator) (Figure 10).

In addition, the xylose operon was composed of the MFS transporter, xylS (alpha-xylosidase [EC 3.2.1.177]) and a transcriptional regulator (Figure 10). All the strains except JHNZZ2M34 contained GH13_29. Meanwhile, JHNZZ2M34 was also the only strain lacking *bglF*. Apart from JHNZZ2M34 and the other 4 exceptions mentioned above, the remaining strains all contained GH13_29 and could utilize xylose.

On the other hand, apart from the glycosyl hydrolases, carbohydrate utilization by *P. pentosaceus* was also related to the isomerase. Among all the 65 strains, 55 strains containing the *araA* gene, which encodes L-arabinose isomerase, were able to grow on medium with L-arabinose as sole carbon source, with three exceptions. At the same time, the remaining strains without *araA* showed no growth on the medium with L-arabinose as sole carbon source. Similarly, there existed consistency between the presence of *rhaA* gene (encoding L-rhamnose isomerase) and the utilization of L-rhamnose (Figure 10).

The cellobiose operon was predicted to be responsible for the transportation and hydrolysis of cellobiose, consisting of *celB* (PTS system, cellobiose-specific IIC component), *bglA* (6-phospho-beta-glucosidase [EC:3.2.1.86], GH1) and *ydhQ* (GntR family transcriptional regulator) (Figure 10). All 65 strains had GH1 family hydrolases and *celB*, which is consistent with their phenotypes since all these strains could utilize cellobiose with one exception, JHZZ2M34, which contained only one GH1 while the other strains contained at least two.

### Prediction of Pediocin and Other Bacteriocins

**FIGURE 11:**
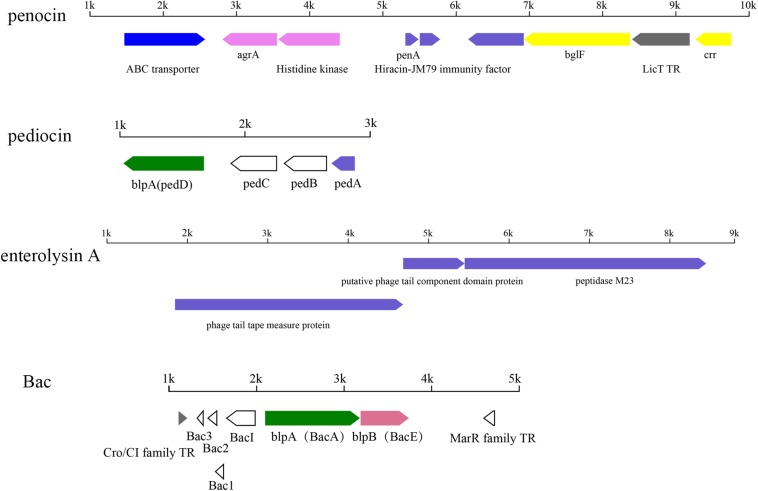
Putative operons for the predicted bacteriocin in *P. pentosaceus.* TR, transcriptional regulator.

In total, four kinds of bacteriocin operon were identified in 54 strains, including two pediocin-like bacteriocins, namely penocin and pediocin, and enterolysin and Bac (Figure 11).

**FIGURE 12:**
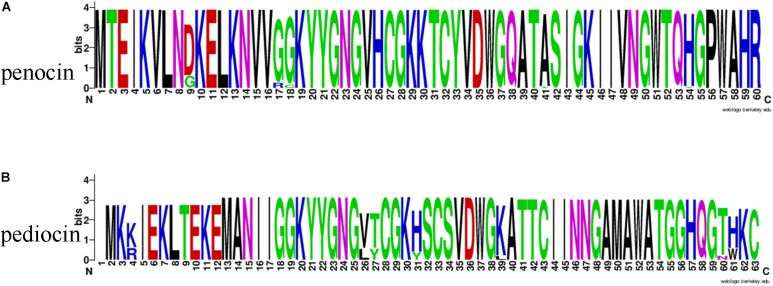
The Weblogo of bacteriocin consensus. **(A)** The Weblogo of penocin consensus. **(B)** The Weblogo of pediocin consensus.

Among all the 54 strains, 35 strains contained *penA* genes encoding penocin, which was annotated as bacteriocin leader domain-containing protein (60aa) in this study (Figure 11). Penocin contained a double-glycine(GG)-leader consensus at its N-terminus (Figure 12A), while the predicted C-terminal mature part had a length of 42 aa and contained the consensus sequence of the pediocin-like bacteriocin family. In addition, gene *penA* was cotranscribed with and/or in close vicinity to a gene encoding Hiracin-JM79 immunity factor (92 aa). In the putative operon, some other proteins involved in the biosynthesis of penocin existed, namely LicT family transcriptional regulator (266 aa), PTS system (encoded by *bglF* and *crr*) and ABC transporter.

Another pediocin-like bacteriocin, pediocin PA-1, was predicted in twelve strains. The operon for pediocin PA-1 consisted of four contiguous genes, namely *pedD*, *pedC*, *pedB*, *pedA* (Figure 11). As the key gene in the operon, *pedA* encoded a highly conserved precursor of pediocin PA-1 (62 aa, Figure 12B), with a YGNGV motif at its N-terminus. The second gene *pedB* was located immediately downstream of *pedA*, and encoded a putative immunity protein (112 aa). The third gene *pedC*, located directly downstream of *pedB*, encoded a probable leucocin-A immunity protein (121 aa), followed by *pedD* (annotated as *blpA* in this study) encoding a Pediocin PA-1 transport/processing ATP-binding protein (214 aa). Upstream of *pedA* existed an acyltransferase (65 aa) and MarR family transcriptional regulator (145 aa), which were inferred to regulate the expression of pediocin PA-1.

Apart from the penocin-like bacteriocin, enterolysin A was also found in 25 strains and annotated as peptidase M23 in this study (Figure 11). Interestingly, according to the annotation against the Swissprot database, these genes were all related to prophage genomes. It was notably that, by comparison of the basic local alignment search tool (BLAST) with the bacteriophages database, gene clusters related to peptidase M23 were homologous to one sequenced bacteriophage predicted in *P. pentosaceus*, namely *Lactobacillus* phage Sha1 (NC_019489).

In addition, the operon of Bac was found in three strains (FSCPS86L4, FSCPS12M2 and FHNXY65L1), encoded by the plasmid in *P. pentosaceus* (Figure 11). The putative operon contained seven genes, with three structural genes codifying the plantaricin (Bac3, Bac2, Bac1), a gene encoding putative bacteriocin immunity protein, a gene encoding ATP-binding protein mesD (LanT), a bacteriocin export accessory protein and a gene encoding putative transcriptional regulator, Cro/CI family.

These four kinds of bacteriocins were not clustered into limited clades on the phylogenetic tree (Figure 5). However, it was shown that, to some degree, penocin might correlate to the evolutionary path, because most of the strains containing genes encoding penocin correlated to clade 1, clade 2, clade 5, and clade 6.

### Prediction of CRISPR/CAS Systems and Prophages in *P. pentosaceus*

**FIGURE 13:**
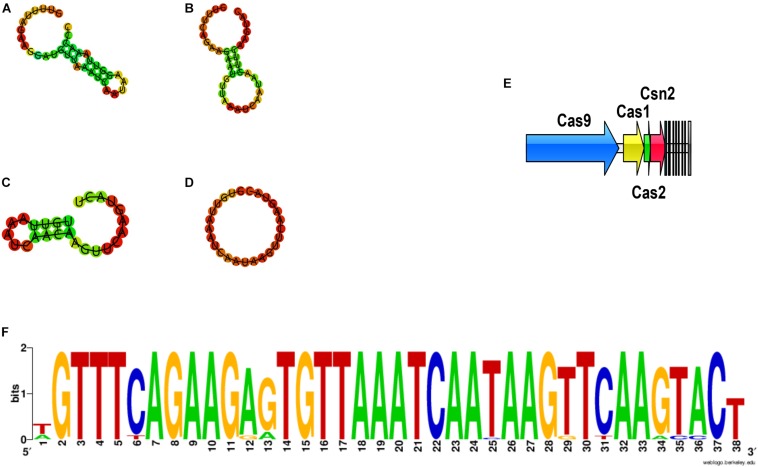
The DR sequences identified in *P. pentosaceus.* **(A)** Secondary structure for DR sequences of Pdpen2 (FHNFQ36L4 and HN26M2); **(B)** Secondary structure for DR sequences of Pdpen3 (JHNZZ2M34, FYNLJ90L2); **(C)** Secondary structure for DR sequences of Pdpen4 (DYNDL69M8); **(D)** Secondary structure for consensus of DR sequences of Pdpen1 (the remaining 27 strains). **(E)** The CRISPR/Cas locus of *P. pentosaceus.* **(F)** The Weblogo of DR sequences within the 32 CRISPR loci.

Analysis of the CRISPR/CAS system was performed for all sequenced genomes (Figure 13). CRISPRs were identified in all the 74 genomes, however, varied in evidence level, and only those whose evidence level was above one were considered in this study. On the other hand, the orphan CRISPRs, without Cas proteins, were ignored due to their inability to silence foreign DNA. In total, 32 strains contained complete CRISPR/CAS systems, and all of those systems belonged to Type IIA, as they contained Cas1, Cas2, Cas9, and Csn2 (Figure 13E), and most of the strains were in the group 1–4 (Figure 5).

CRISPRs were mainly defined by the Direct Repeat (DR) sequences, and the typical DR sequences were defined as the most frequent sequence within a particular CRISPR locus. Therefore, it was unsurprising to find that all the DR sequences within the 32 CRISPR loci showed highly similarity (Figure 13F). To confirm which CRISPR repeat family the DR sequences in *P. pentosaceus* belong to, multiple sequence alignments and phylogenic tree were performed for DR sequences from *P. pentosaceus* and other sequences from eight different families. Pdpen1 represented the consensus sequence in 26 strains where each base corresponded to the most frequent nucleotide at each position, and the other six genomes (FSCDJY41L1, FSCCD15L1, FSDHZD6L3, DYNDL69M8, JHNZZ2M34, FHNFQ36L4) represented sequences that harbored slight differences in some position, respectively. In light of the phylogenic tree, all the DR sequences from *P. pentosaceus* belonged to the Lsal1 family (Horvath et al., 2009) (Supplementary Figure S1). In addition, to depict the RNA secondary structure and MFE (minimum free energy) of the DR sequences, all the sequences were analyzed through the RNA fold web server (Table 2 and Figure 13). According to the secondary structures, all the sequences were divided into 4 groups, namely Pdpen1, Pdpen2, Pdpen3, and Pdpen4. The secondary structure of Pdpen1 represented the most common structures in the *P. pentosaceus* DR sequences, and was a large loop without stem. Furthermore, the MFE of Pdpen1 was the largest among all the groups (Table 2 and Figure 13D). On the other hand, RNA secondary structures of Pdpen2, Pdpen3, and Pdpen4, all contained two rings at both ends with a stem in the middle, of which the MFEs were −1.6 kcal/mol, −0.3 kcal/mol, and −0.1 kcal/mol, respectively (Table 2 and Figures 13A–C). Noticeably, on the stem, Pdpen2 also had a small loop and G:U base pairs, which was typical of conserved RNA secondary structures and attached importance to the stem-loop in the repeats for the functionality of CRISPRs. Notably, Pdpen1 CRISPRs may or may not be active since the secondary structure and the MFE have not been reported elsewhere. More research is warranted to further define Pdpen1. However, the CRISPR array could still be useful to analyze the potential evolutionary path and genomic diversity of *P. pentosaceus*.

**TABLE 2:** Classification for the DR sequences.

Secondary structure	Strain	Consensus direct repeat sequence	Minimum free energy (MFE) / kcal/mol
Non-circular	FHNFQ36L4	GTTTTAGAAGGATGTTAA ATCAATAAGGTTAAACCC	−1.6
	HN26M2_2		
	JHNZZ2M34	GTTTCAGAAGAATGTTAA ATCAATAAGTTCAAGTAC	−0.3
	FYNLJ90L2		
	DYNDL69M8	TGTTAAATCAA CAAGTTCAAGTACT	−0.10
Circular	27 strains	TGTTAAATC AATAAGTTCAAGTAC	0

In total, 349 spacers were found in 32 strains of *P. pentosaceus*. The sequences of the spacers in each locus showed high diversity.

**FIGURE 14:**
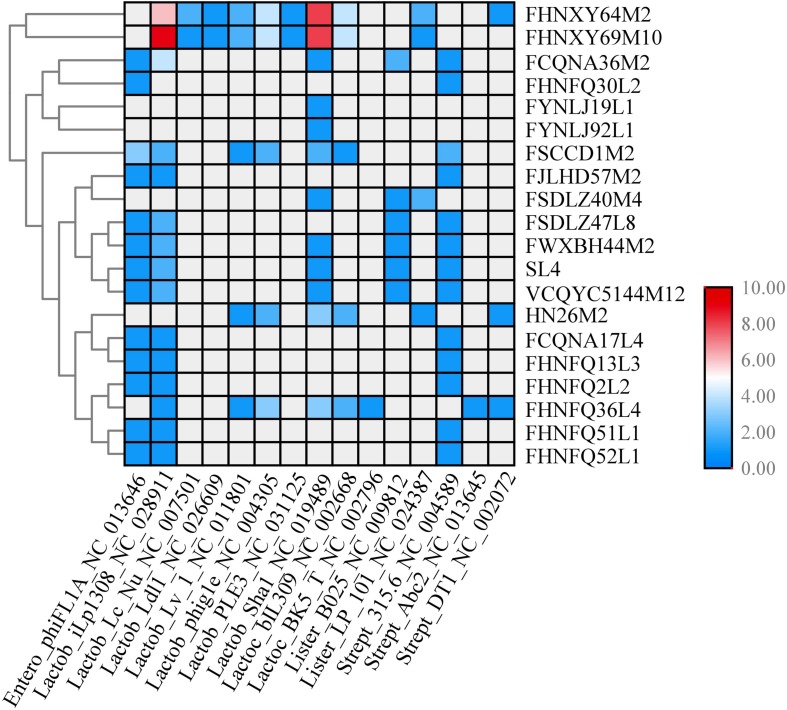
CRISPR spacers targeting prophages in *P. pentosaceus* genomes. The heatmap represents *P. pentosaceus* CRISPR spacers that matched prophages in *P. pentosaceus*. The vertical axis represents the genomes that harbor prophages targeted by *P. pentosaceus* CRISPR spacers. The horizontal axis represents *P. pentosaceus* strains carrying CRISPR spacers that target prophages. The color scales represent the number of targeting events with gray squares represents the absent of matches and red squares representing the highest number of targeting.

To clarify the possible immunity backgrounds and evolutionary path of *P. pentosaceus*, all of the 349 spacers found in the CRISPR loci were analyzed using Blastn along with potential prophages present in the genomes. PHAST was used to predict prophage sequences, and a total number of 21 kinds of prophages were predicted in 57 strains with only intact prophages considered in this study (Supplementary Table S3). The number and types of prophages may not be confined to a particular phylogenetic group of bacterial strain and even closely related isolates may differ in the number and kinds of prophages they harbor. From 349 spacers, 84 spacers were selected based on the following criteria: identity, 100%; number of mismatches, 0; *e*-value less than 7.00E-11; and alignment length more than 30 bp. Eighty four spacers corresponding to 21 strains were homologous to a total number of 19 phage genomes (Figure 14).

**FIGURE 15:**
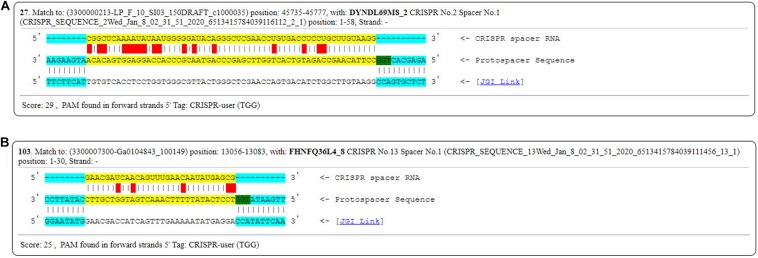
PAMs sequences found in DYNDL69M8 and FHNFQ36L4. **(A)** PAMs sequences found in DYNDL69M8. **(B)** PAMs sequences found in FHNFQ36L4.

In order to investigate the possible activity of the spacers, PAMs were identified from the six genomes mentioned above (FSCDJY41L1, FSCCD15L1, FSDHZD6L3, DYNDL69M8, JHNZZ2M34, FHNFQ36L4). PAM sequence 5′-TGG-3′ from DYNDL69M8 and FHNFQ36L4 was detected (Figure 15). This PAM sequence is homologous to the 5′-NGG-3′ previously established for *Streptococcus pyogenes* Cas9 (Hsu et al., 2013).

## Discussion

*Pediococcus pentosaceus*, as a reliable bacteriocin producer, has gained much interest to prevent the growth of pathogenic and spoilage bacteria during food fermentation and preservation (Zommiti et al., 2019). Furthermore, it has been reported that *P. pentosaceus* can stimulate the mammalian immune system, reduce total and LDL cholesterol levels and improve protein digestion (de Azevedo et al., 2019). With the development of comparative genomics, it has become possible to predict microbial functions before conducting animal experiments. However, current available genomes of *P. pentosaceus* are limited and not diverse enough for comparative genomic analysis. In this study, 65 strains of *P*. *pentosaceus*, previously isolated from different niches in China, were draft genome sequenced and analyzed using a comparative genomics approach to reveal genomic diversity and distribution in this species.

The average G + C% content and genome size were ∼37.26% and ∼1.86 Mbp, respectively, which were not only consistent with previous results for *P. pentosaceus* strains (Jetten et al., 2015), but also in line with such characteristics of lactobacilli (Makarova et al., 2006; Zheng et al., 2015). In addition, since dispensable genes contribute to species diversity and might encode supplementary biochemical pathways and functions that are essential for selective advantages (Medini et al., 2005), the wide range of total CDSs predicted per genome (from 1692 to 2170) and the large proportion of variable genes (78%) both indicate a comparatively high level of diversity (Arboleya et al., 2018). Unprecedentedly, a pan-genome of 73 genomes described in this work, displayed a trend of not fully closed but approaching closure, and revealed the core genes of these strains mostly encoded functions related to translation, ribosomal structure and biogenesis, signal transduction mechanisms, and general function prediction only. Apart from that, it is worth mentioning that through whole genome comparisons, we found genes predicted to represent mobile genetic elements (e.g., plasmids), phage proteins as well as a considerable amount of hypothetical proteins. This also suggests that horizontally transferred DNA plays an important role in genome diversity in this species (Arboleya et al., 2018), indeed, several publications mention that *P. pentosaceus* contains plasmids belonging to other species or even genera (Kantor et al., 1997; Miller et al., 2005).

The phylogenetic tree based on sequence similarity of orthologous genes grouped 74 *P. pentosaceus* strains into six clades, with no apparent correlation between the branches and isolation sources, while the genotypic methods applied to these strains yielded results that appeared to be very homogeneous (e.g., characteristics related to CRISPR/Cas, bacteriocin production). Meanwhile, as reported in previous publications, some phenotypic traits, such as characteristics related to growth, the ability to acidify and carbohydrate metabolism, might be homogenous in some cases (Martino et al., 2013). Therefore, another phylogenetic tree based on a smaller number of strains was performed with the public genomes excluded, in order to match genotype to phenotype on metabolizing carbohydrates. Unfortunately, the current results failed to elucidate the correlation between the clades and evolutionary relationship among these strains. Although the strains were enough, especially with different origins, it was difficult to show the exact evolutionary path of the species at a certain extent, in that *Pediococcus*, as a sub-clade of lactobacilli, can be ‘allochthonous,’ and has neither an ecological nor evolutionary relationship with the habitat where they occur (Duar et al., 2017). For example, in the sixth group of the phylogenetic tree, *P. pentosaceus* FHNFQ30L2 and HN25-2 isolated from human fecal samples were clustered with the remaining strains that were all isolated from fermented food products (DYNDL- from dairy fan, JHNZZ2M34 from “emptins”), from which it could be inferred that strains in the human gastrointestinal tract can originate from fermented food. In addition, the results from the multiple gene alignments also supported the argument, as FSCPS12M2 and FSCPS86L4 were determined to be from the same source, even though they were isolated from completely different niches in the same sampled region. Therefore, further investigation on the real ecological niches and the exact evolutionary path of *P. pentosaceus* needs further investigation.

The carbohydrate utilization ability of bacteria is an important indicator of strain functionality, and lays a foundation for further cultivation and selection of the strain. Twenty-three carbohydrate fermentations for the 65 strains were determined, and did not differentiate the strains based on their sampled origin or lineage that grouped on the phylogenetic tree, probably because the phenotypic behavior depends on the genes presenting on mobile elements. Notably, 40 strains were able to utilize D-lactose or L-lactose as the sole carbon source, which is contradictary to the view that lactose-positive strains within the naturally occurring *Pediococcus* genus are absent (Semjonovs and Zikmanis, 2008). Hence, those *P. pentosaceus* strains might be acceptable starters for dairy fermentation. Actually, it has been reported that pediococci can increase the positive organoleptic characteristics of cheese through catabolism of lactate (Thomas et al., 1985), and *P. pentosaceus*, in particular, was able to increase antioxidant activity and fatty acid profile of fermented milks, in order to enhance the therapeutic value of the products (Balakrishnan and Agrawal, 2014). Besides, in the current study, seven putative operons related to the utilization of sucrose, D-cellobiose, D-lactose and L-lactose, D-xylose, D-trehalose, L-arabinose, and L-rhamnose were analyzed, in which four glycosyl hydrolases and two isomerases were identified to be the key enzymes in the corresponding operons (O’Donnell et al., 2011). Specifically, genes *sacA*, *bglA*, *lacZ*, *xylS*, and *treC* encoded key enzymes for utilizing sucrose, cellobiose (Bai et al., 2017), lactose (Wheatley et al., 2013), xylose and trehalose (Schluepmann et al., 2003). It’s worth noting that gene *sacA* was also important in FOS metabolism (Chen et al., 2014), and it’s possible that the utilization of FOS was controlled by the same operon involved in sucrose utilization (data not shown). In addition, gene *araA* and *rhaA* encoded key isomerases involved in utilizing L-arabinose and L-rhamnose, respectively (Arzamasov et al., 2018; Regmi and Boyd, 2019). Most of the strains that failed to utilize these carbohydrates lacked genes encoding key enzymes and isomerases. Sugar transport in bacteria is catalyzed by ABC-binding cassette (ABC) transporters (Schneider, 2001), secondary carriers and phosphotransferase systems (PTS) (Erni, 2012; Saier, 2015). It is notable that those five operons distinguished by glycosyl hydrolases were all co-transcribed with different PTS systems, while the latter two operons that used isomerases as key enzymes for carbohydrate metabolism were co-transcribed with the MFS system, as in the D-xylose proton-symporter. In the analysis of gene-trait-matching, the lack of specific transporter system was another reason explaining the mix-matches in the exceptions, such as JHZZ2M34, which failed to metabolize trehalose due to the lack of *bglF* and GH13_29. Furthermore, gene re-arrangements incapacitating metabolism of some carbohydrate existed in *P. pentosaceus* as well. For example, JHNZZ2M34 harbored both *celB* and *bglA* but which were not contiguous, and was the only strain that could not utilize cellobiose, indicating that *celB* and *bglA* might need to be contiguous and probably co-transcribed if they are to be active in the hydrolysis of cellobiose, which corresponds to the genetic organization of the cellobiose locus in *Streptococcus mutans* (Zeng and Burne, 2009).

Various strains of *P. pentosaceus* have been reported to produce bacteriocins such as pediocin PA-1 (Marugg et al., 1992), pediocin ST18 and pediocin-A (Jetten et al., 2015), and the ability to produce such bacteriocins has attracted much attention. In the current work, four kinds of bacteriocins were found in these strains, of which two bacteriocins were not reported in the species before, namely enterolysin A and Bac. Consistently, it was found that genes encoding enterolysin A were homologous with predicted prophage associated genes, and genes encoding Bac were identified on plasmids in the strains. Although in this study potential plasmids were not analyzed, pediocin A-1 genes have been reported to be plasmid-associated, and encoded by highly similar operons on these plasmids (Miller et al., 2005). Therefore, it is possible that *P. pentosaceus* seized the ability to produce enterolysin A and Bac from other species, as none of these bacteriocins are reportedly encoded by *P. pentosaceus*, and both of these bacteriocin operons are placed on mobile elements. It has also been reported that several *P. pentosaceus* strains harbor plasmids which contain cassettes commonly found in other lactic acid bacteria (Alegre et al., 2005; Teresa Alegre et al., 2009), which is consistent with the above observation. This provides a new perspective for the study on evolutionary relationships among species.

CRISPR in combination with Cas constitute CRISPR-Cas systems, which provide adaptive immunity against invasive elements in bacteria (Barrangou et al., 2007). In fact, it has been mentioned that most lactobacilli contain type II CRISPR/Cas, and is in line with *P. pentosaceus* strains analyzed here which belong to the subclade of lactobacilli (Sun et al., 2015). However, we observed that 32 strains of all the 74 strains contained type IIA CRISPR/Cas. The distribution of these strains on the phylogenetic tree revealed the possible correlation between the immunity system against external genetic material and the evolutionary path. That the DR sequences clustered in the Lsal1 family indicates the close relationship between *Pediococcus* and *Lactobacillus*, corresponding to the view that horizontal transfer dominates the evolution of CRISPR/Cas loci (Horvath et al., 2009; Makarova et al., 2015a). Additionally, as to the four kinds of secondary structure of DR sequences, the RNA secondary structure reflected the conservation of the repeat sequences, and the presence of the G:U base pair highlighted the importance and the stability of this structure (Scaltriti et al., 2019). Thus, it is possible that the disability of Pdpen1 to form a stem-loop revealed the variability of the array and might correlate to the functionality of CRISPRs. In addition, as a discriminatory tool, spacers in the array have been strongly associated with sequence-based detection (Fricke et al., 2011), and showed a degree of homology with prophages predicted in the 74 strains, which indicates that they were probably result from diverse events involving phage infection. Besides, the PAM sequences found in DYNDL69M8 and FHNFQ36L4 could be implicated in novel spacer acquisition (Paez-Espino et al., 2013), and Cas9-mediated sequence-specific cleavage of target sequences (Sapranauskas et al., 2011; Gasiunas et al., 2012; Karvelis et al., 2013). However, it is notable that validation of CRISPR/Cas needs future experimental evidence to further determine activity.

## Conclusion

In this study, the genomes of 65 strains of *P. pentosaceus* were sequenced and analyzed through a comparative genomics approach. The results showed that *P. pentosaceus* is a non-host/non-niche-specific species. The genomes mainly differed in carbohydrate metabolism, and horizontally transferred DNA, such as bacteriocins encoded by plasmids and prophages present. Additionally, the CRISPR/Cas system was predicted to be of type IIA. The genome sequencing and the genetic analyses conducted in this study allow for a deeper understanding of the biotechnological potential of *P. pentosaceus*, facilitating its future development as additives/starters for the dairy industry and therapeutics for microbiota-related diseases. However, there are still limitations in this article thus future studies will focus on delineating the interactions between the host and *P. pentosaceus*, and further elucidating the genomic correlation between adaptation and phenotypes.

## Data Availability Statement

The datasets generated for this study can be found in the https://www.ncbi.nlm.nih.gov/bioproject/PRJNA580075.

## Author Contributions

JJ, BY, WC, RR, and CS designed the research. JJ carried out the experiments. JJ, BY, JZ, and HZ analyzed the data. HZ and WC contributed reagents, materials, and analysis tools. JJ and BY wrote the manuscript. CS, RR, HZ, and WC revised the manuscript. All the authors had read and approved the final manuscript.

## Conflict of Interest

The authors declare that the research was conducted in the absence of any commercial or financial relationships that could be construed as a potential conflict of interest.

## Footnotes

1 https://www.ncbi.nlm.nih.gov/genbank

2 https://github.com/widdowquinn/pyani

3 https://github.com/jvollme/fasta2phylip

4 http://www.evolgenius.info/evolview/

5 https://crisprcas.i2bc.paris-saclay.fr/CrisprCasFinder/Index

6 http://crispr.otago.ac.nz/CRISPRTarget/crispr_analysis.html

7 http://www.cazy.org/

## Supplementary Material

FIGURE S1 Phylogenic tree of DR sequences from *P. pentosaceus* and other eight families.

TABLE S1 The assembly parameters of 65 *P. pentosaceus* strains.

TABLE S2 The annotation of the referenced genome *P. pentosaceus* ATCC25745.

TABLE S3 Prophages predicted in 57 *P. pentosaeus* strains using PHAST.
