# Supplementary figures and images for: Comparative Genomics of Pediococcus pentosaceus Isolated From Different Niches Reveals Genetic Diversity in Carbohydrate Metabolism and Immune System
Source: Front Microbiol. 2020 Feb 26;11:253. doi: 10.3389/fmicb.2020.00253 (PMC7055311; doi:10.3389/fmicb.2020.00253)

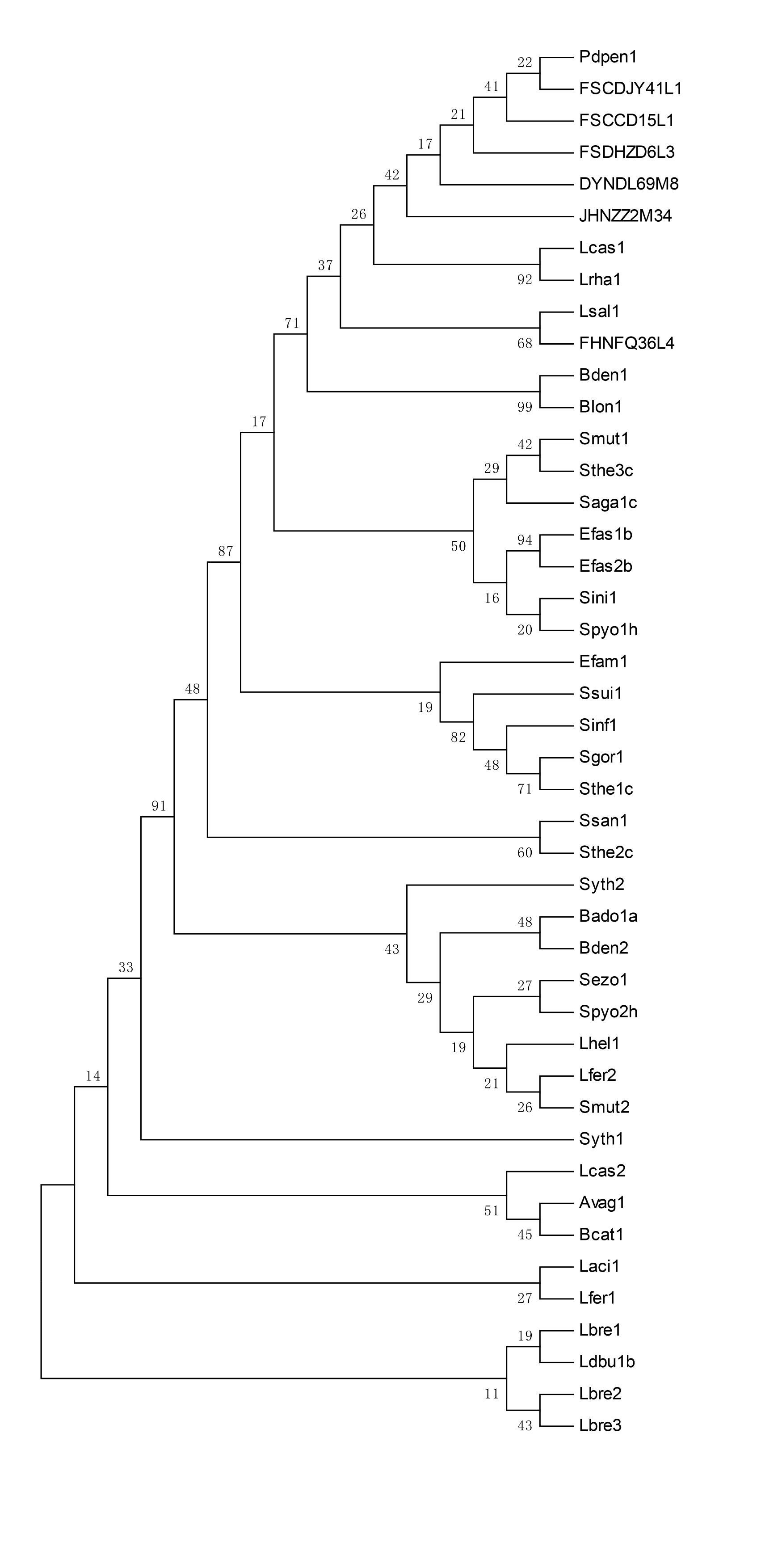

Supplement: FIGURE S1 — Phylogenic tree of DR sequences from P. pentosaceus and other eight families. [file Image_1.TIF]
